# Factors Influencing HIV Drug Resistance among Pregnant Women in Luanda, Angola: Findings from a Cross-Sectional Study

**DOI:** 10.3390/tropicalmed6010029

**Published:** 2021-03-05

**Authors:** Cruz S. Sebastião, Joana Morais, Miguel Brito

**Affiliations:** 1 Centro de Investigação em Saúde de Angola, Caxito, Angola; cruzdossantos10@gmail.com (C.S.S.); jmafonso.7@gmail.com (J.M.); 2 Molecular Biology Laboratory, Instituto Nacional de Investigação em Saúde, Luanda, Angola; 3 Instituto Superior de Ciências da Saúde, Universidade Agostinho Neto, Luanda, Angola; 4 Faculdade de Medicina, Universidade Agostinho Neto, Luanda, Angola; 5 Health and Technology Research Center, Escola Superior de Tecnologia da Saúde de Lisboa, Instituto Politécnico de Lisboa, 1990-096 Lisboa, Portugal

**Keywords:** HIV infection, antiretroviral failure, risk factors, pregnant women, Angola

## Abstract

The increase in HIV infection and drug-resistant strains is an important public health concern, especially in resource-limited settings. However, the identification of factors related to the propagation of infectious diseases represents a crucial target offering an opportunity to reduce health care costs as well as deepening the focus on preventing infection in high-risk groups. In this study, we investigate the factors related to drug resistance among HIV-infected pregnant women in Luanda, the capital city of Angola. This was a part of a cross-sectional study conducted with 42 HIV-positive pregnant women. A blood sample was collected, and HIV-1 genotyping was carried out using an in-house method. Multivariate analyses were performed to determine the interaction between sociodemographic characteristics and drug resistance. HIV drug resistance was detected in 44.1% of the studied population. High probabilities of drug resistance were observed for HIV-infected pregnant women living in rural areas (AOR: 2.73; 95% CI: 0.50–14.9) with high educational level (AOR: 6.27; 95% CI: 0.77–51.2) and comorbidities (AOR: 5.47; 95% CI: 0.28–106) and infected with a HIV-1 non-B subtype other than subtype C (AOR: 1.60; 95% CI: 0.25–10.3). The present study reports high HIV drug resistance. Furthermore, older-age, rural areas, high educational levels, unemployed status, having comorbidities, and HIV-1 subtypes were factors related to drug resistance. These factors impact on drug susceptibility and need to be urgently addressed in order to promote health education campaigns able to prevent the spread of drug-resistant HIV strains in Angola.

## 1. Introduction

The human immunodeficiency virus (HIV) remains a global public health problem [1]. In 2019, an estimated 37.9 million people were living with HIV worldwide [2]. Of these, around 220,000 of the infected were living in Angola [2]. The HIV infection is caused by two lentiviruses (HIV-1 and HIV-2) divided into groups (M-P), subtypes (A–D, F–H, J, and K), sub-subtypes (A1, A2, F1, and F2), and circulating recombinant forms (CRFs) [3].

The antiretroviral drugs currently approved to treat HIV infection belong to distinctive classes that act in different phases of the HIV replication as fusion inhibitor, nucleoside reverse transcriptase inhibitors (NRTIs), non-nucleoside reverse transcriptase inhibitors (NNRTIs), integrase inhibitors, and protease inhibitors (PIs) [4].

Countless factors, including drug-resistant strains, HIV subtypes, viral load monitoring, treatment adherence, comorbidities, and sociodemographic characteristics, have been associated with the development of drug resistance in HIV-infected pregnant women from low- and middle-income countries (LMICs) [5,6,7,8,9,10].

The identification of factors related to HIV drug resistance (HIVDR) represents a crucial goal to ensure the effectiveness of antiretroviral treatment (ART). To the best of our knowledge, this is the first study that investigates factors related to the spread of drug resistance mutation (DRMs) in HIV-positive pregnant women in Luanda, the capital of Angola. The information set out in this paper is an extension of our recently published study on HIV-1 diversity and drug resistance in pregnant women [11]. Hence, this study makes it possible to promote health education campaigns and strengthen the ongoing strategies in the control of HIVDR in Angola.

## 2. Materials and Methods

### 2.1. Study Design and Setting

This was part of a cross-sectional study carried out by the same research team with 42 HIV-infected pregnant women, recently diagnosed and without prior exposure to ART, between April and June 2018, at the Lucrecia Paim Maternity Hospital in Luanda, the capital of Angola. This is the largest public maternity unit and a reference center for maternal health care, training, and research in Angola. Furthermore, it is a tertiary health institution which provides health care for pregnant women and newborns from all national provinces. The main study inclusion criteria for pregnant women were confirmed diagnosis of HIV infection and no ART exposure. The study protocol was reviewed and approved by the Angolan National Ethics Committee (nr.13/2018) and the General Directorate of Lucrecia Paim Maternity, Luanda, Angola (nr.083/GDG/MLP/2018). The study was verbally explained to the participants and with written informed consent obtained from all participants, or the parents/legal guardians of minors aged under 15, before enrolment in the study.

### 2.2. Data Collection and Procedure

The research team collaborated with the hospital’s teams for data collection. A structured questionnaire served to obtain sociodemographic characteristics, age, place of residence, level of education, occupation, and presence of comorbidities (Supplementary Material). For each participant, a blood sample was collected using vacutainers, and all prepared serum samples were stored at −80 °C until usage. HIV-1 genotyping test was carried out according to an in-house method as previously described [11]. The HIV nucleotide sequences obtained were submitted to the GenBank (NCBI) database and assigned accession numbers MK543512 to MK543545. HIV subtypes were confirmed by the REGA HIV-1 Subtyping Tool [12]. The identification of HIVDR was assessed with the genotypic resistance interpretation algorithm implemented in the Stanford HIV drug resistance database website (https://hivdb.stanford.edu/hivdb/by-sequences/ accessed on 3 September 2018) [13].

### 2.3. Statistical Analysis

Statistical analyses were performed by SPSS v25 (IBM SPSS Statistics, Chicago, IL, USA). Categorical variables are presented as frequencies and percentages, and continuous variables as means and standard deviations (SD). Factors related to HIVDR were assessed using a logistic regression model. Univariate and multivariate analysis was performed with all independent variables and an outcome variable. All independent variables were included in the multivariate analysis to ensure that all potentially important variables were maintained. The goodness of fit for the model was verified by the Hosmer–Lemeshow test. The strength and direction of the relationship were determined with an adjusted odds ratio (AOR) and their 95% confidence interval (CI). All reported *p*-values are two-tailed with a level of significance set at 5%.

## 3. Results

All 42 pregnant women consented to participate in the study, provided the demographic data, and a blood sample for laboratory tests. Analysis of the HIV subtypes and DRMs could only be obtained in 34 out of the 42 samples subjected to HIV-1 genotyping. This was not possible in the remaining samples even after countless repeated attempts applying different conditions and PCR primers. Thus, only data on the 34 pregnant women successfully amplified and genotyped were included in the analyses.

Of the 34 samples genotyped, 15/34 (44.1%) presented DRMs. Out of these 15 pregnant women with mutations, 14/15 (93.3%), 2/15 (13.3%), and 1/15 (6.7%) presented resistance against the NNRTIs, NRTIs, and PIs, respectively. Furthermore, 2/15 (13.3%) samples reported multidrug-resistance, one to NRTIs/NNRTIs and the other to PIs/NNRTIs. The mutations E44ED, M41L, D67N, T69D, and T215S were detected in the NRTIs, whereas the mutations V179E, E138A, V108I, V106I, G190A, P225H, K103N, Y181I, K103Q, and E138G were detected in the NNRTIs. The mutation L33F was the only one detected in the PIs (Table 1).

All pregnant women were infected with the non-B subtype (nBS) from HIV-1 group M. Of these, subtype C (*n* = 13, 38.2%) was the most frequent. Furthermore, subtypes F1, A1, G, D, H, F1/C, A1/G, H/G, CRF02_AG, and CRF37_cpx were found.

The putative factors related to HIVDR are set out in Table 2. The age range was 19 to 42 years. The mean age was 29 ± 6. Most of the pregnant women (*n* = 28, 82.4%) were aged over 24 years, with high educational levels (*n* = 21, 61.8%), employed (*n* = 19, 55.9%), without comorbidities (*n* = 29, 87.9%), and infected with HIV-1 nBS other than subtype C (*n* = 21, 61.8%). The analysis returned no significant relationship (*p* > 0.05) and, even so, the multivariate analytical findings demonstrate that the likelihood of developing resistance in pregnant women from rural areas was 2.7 times (95% CI: 0.50–14.9), with a high educational level was 6.3 times (95% CI: 0.77–51.2), with comorbidities was 5.5 times (95% CI: 0.28–106), and with HIV-1 nBS other than subtype C was 1.6 times (95% CI: 0.25–10.3). On the other hand, pregnant women aged under 25 (AOR: 0.28 (95% CI: 0.03–2.91), *p* = 0.287) and employed pregnant women (AOR: 0.32 (95% CI: 0.05–2.10), *p* = 0.235) were potential protective factors (Table 2).

## 4. Discussion

Access to ART is important for reducing mortality and prevents HIV transmission [14,15]. These public health accomplishments were made possible by the widespread administration of standardized ART regimens characterized by an inexpensive fixed-dose combination of NRTIs plus NNRTIs [16]. Although efforts are being made to control HIV, infected people can acquire DRM and can also be infected with drug-resistant strains [17].

In this study, we report a small study with ART-naïve pregnant women in terms of the burden of transmitted DRMs, and to investigate factors related to increasing HIVDR. We found a high (≥15%) drug resistance level according to the WHO [18]. The resulting resistance level was thus higher than those reported in pregnant women from Ghana (<5%) [5], Nigeria (<5%) [7], Malawi (7.6%) [8], and Guinea Bissau (10.4%) [10], while lower than that returned in Tanzania (97%) [9]. The drug resistance level observed in these pregnant women represents a threat to Angola’s ART program [19,20].

The identification of the sociodemographic factors of the HIV-affected population is a crucial target for intervention and control of the emergence of DRMs. To the best of our knowledge, this is the first study on factors related to the scatter of HIVDR among pregnant women from Angola. Age, patient residence, educational level, occupation, comorbidities, and HIV-1 subtypes were found to be factors related to the risk of ART failure supported by results recorded by previous studies in LMICs [5,6,7,8,9,10].

Consistent with our findings, Wilhelmson et al. [10] and Imade et al. [7] reported a high frequency of DRMs in pregnant women aged over 24 years in Guinea Bissau and Nigeria, respectively. Our results are also similar to the findings of Khienprasit et al. [21], which reported a lower likelihood of developing treatment failure in HIV-positive women from urbanized areas, whereas the HIV-positive pregnant women with comorbidities (OR = 1.49 (95% CI = 1.03–2.15), *p* = 0.032) were associated with treatment failure. By contrast, Chagomerana et al. [8] identified how HIV-positive pregnant women with high educational levels were less likely to develop treatment failure, while Wilhelmson et al. observed unfavorable virologic outcomes in pregnant women with low educational level [10].

The protective factor observed in pregnant women aged under 25 years was consistent with studies performed by Khienprasit et al. [21] and Chao et al. [22]. By contrast, Crabtree-Ramírez et al. [23] reported higher odds related to ART failure in young women (OR: 4.7, (95% CI: 1.5–14.4), *p* = 0.003). On the other hand, the protective factor for employed pregnant women was consistent with a study carried out by Bayu et al. [24].

Based on these results, a focus on younger women, employed and from urbanized areas, should be prioritized to ensure better ART outcomes. Additionally, it is urgent to increase the screening for comorbidities and points of care to detect ART failure in rural areas.

Studies showed improved virological outcomes in patients with nBS [25]. However, a widely discussed issue is the impact of ART on nBS once drug design has been performed on subtype B and extended to nBS [26]. Herein, we detect a high likelihood of present DRM in women with other nBS compared to subtype C (AOR: 1.60) (Table 2). For this reason, an update of the algorithms to interpret HIVDR and assess the drugs to which their virus is susceptible should be considered prior to the selection of ART regimens [27].

Our study has limitations. The small sample size limits the conclusions of our study. Besides, this study does not represent the whole population of pregnant women from Luanda or other regions from Angola. The HIV viral load, CD4 count, and other clinical factors were not determined in the enrolled subjects due to a lack of laboratory resources. A low viral load or the high genetic diversity of HIV-1 subtypes circulating in Luanda may explain the failure of the amplification of the viral RNA of the remaining eight samples. Further studies with a larger sample size and participants from other communities in Angola are needed to elucidate the burden of HIVDR in Angolan women. Furthermore, in settings with a poor roll-out of virological load monitoring, the investigation of the factors related to drug resistance will generate a great impact on the development of more effective public health strategies to control the HIV epidemic in LMICs.

## 5. Conclusions

Our findings show that HIV drug resistance is a public health burden among pregnant women in Luanda that needs addressing. Older-aged, rural areas, a high educational level, unemployed, comorbidities, and HIV-1 non-B subtypes were factors related to the increase in drug resistance in pregnant women from Luanda. Therefore, if the risk factors related to the spread of HIV drug resistance are not urgently addressed, they may increase hard-to-treat infections and health care costs in Angola.

## Figures and Tables

**Table 1:** Drug resistance mutations to nucleoside reverse transcriptase inhibitors (NRTIs), non-nucleoside reverse transcriptase inhibitors (NNRTIs), and protease inhibitors (PIs) according to the HIV-1 subtypes.

HIV-1 Subtypes	HIV-1 Drug Resistance Mutation		
	NRTI	NNRTI	PI
F1	-	V179E	-
F1/C	-	E138A	-
C	-	V108I	-
A1/G	-	V106I; G190A	-
F1	-	P225H; K103N; E138A	-
C	-	E138A	-
C	-	E138A	-
D	E44ED	-	-
H	-	G190A	-
G	M41L; D67N; T69D; T215S	Y181I	-
C	-	E138A	-
CRF37_cpx	-	E138A	-
A1	-	K103Q	L33F
A1	-	E138G	-
C	-	K103N	-

**Table 2:** Putative factors related to drug resistance among HIV-positive pregnant women in Luanda, Angola, 2018.

Characteristics	*n* (%)	Resistance to NNRTIs		Resistance to NRTIs		Resistance to PIs		Univariate Analysis		Multivariate Analysis *	
		No (%)	Yes (%)	No (%)	Yes (%)	No (%)	Yes (%)	OR (95% CI)	*p*-Value	AOR (95% CI)	*p*-Value
Overall	34 (100)	20 (58.8)	14 (41.2)	32 (94.1)	2 (5.9)	33 (97.1)	1 (2.9)				
Age groups ^a^											
<25 years	6 (17.6)	4 (66.7)	2 (33.3)	6 (100)	0 (0.0)	6 (100)	0 (0.0)	0.58 (0.09–3.68)	0.561	0.28 (0.03–2.91)	0.287
≥25 years	28 (82.4)	16 (57.1)	12 (42.9)	26 (92.9)	2 (7.1)	27 (96.4)	1 (3.6)	1.00	-	1.00	-
Place of residence ^b^											
Urban area	17 (50.0)	12 (70.6)	5 (29.4)	16 (94.1)	1 (5.9)	16 (94.1)	1 (5.9)	1.00	-	1.00	-
Rural area	17 (50.0)	8 (47.1)	9 (52.9)	16 (94.1)	1 (5.9)	17 (100)	0 (0.0)	2.06 (0.52–8.18)	0.303	2.73 (0.50–14.9)	0.247
Education ^c^											
Low	13 (38.2)	10 (76.9)	3 (23.1)	12 (92.3)	1 (7.7)	13 (100)	0 (0.0)	1.00	-	1.00	-
High	21 (61.8)	10 (47.6)	11 (52.4)	20 (95.2)	1 (4.8)	20 (95.2)	1 (4.8)	2.48 (0.58–10.6)	0.223	6.27 (0.77–51.2)	0.086
Occupation ^d^											
Unemployed	15 (44.1)	8 (53.3)	7 (46.7)	14 (93.3)	1 (6.7)	14 (93.3)	1 (6.7)	1.00	-	1.00	-
Employed	19 (55.9)	12 (63.2)	7 (36.8)	18 (94.7)	1 (5.3)	19 (100)	0 (0.0)	0.51 (0.13–2.02)	0.339	0.32 (0.05–2.10)	0.235
Comorbidities ^e,#^											
No	29 (87.9)	19 (65.5)	10 (34.5)	27 (93.1)	2 (6.9)	28 (96.6)	1 (3.4)	1.00	-	1.00	-
Yes	4 (12.1)	1 (25.0)	3 (75.0)	4 (100)	0 (0.0)	4 (100)	0 (0.0)	4.91 (0.45–53.3)	0.191	5.47 (0.28–106)	0.261
HIV-1 nBS ^f^											
Subtype C	13 (38.2)	8 (61.5)	5 (38.5)	13 (100)	0 (0.0)	13 (100)	0 (0.0)	1.00	-	1.00	-
Others nBS	21 (61.8)	12 (57.1)	9 (42.9)	19 (90.5)	2 (9.5)	20 (95.2)	1 (4.8)	1.46 (0.36–5.95)	0.602	1.60 (0.25–10.3)	0.619

Abbreviations: nBS, non-B subtypes; NNRTIs, non-nucleoside reverse transcriptase inhibitors; NRTIs, nucleoside reverse transcriptase inhibitors; PIs, protease inhibitors; OR, odds ratio and their 95% confidence interval (CI); AOR, adjusted odds ratio. Reference class: ^a^ ≥25 years old; ^b^ Urban area; ^c^ Low education; ^d^ Unemployed; ^e^ No comorbidities; ^f^ Subtype C. ^#^ Missing value = 1. * Adjusted for all the independent variables listed.

## Data Availability

The datasets generated during and/or analysed during the current study are available from the corresponding author on reasonable request.

## Supplementary Materials

The following are available online at https://www.mdpi.com/2414-6366/6/1/29/s1.
