# Supplementary material for: Factors Influencing HIV Drug Resistance among Pregnant Women in Luanda, Angola: Findings from a Cross-Sectional Study
Source: Trop Med Infect Dis. 2021 Mar 5;6(1):29. doi: 10.3390/tropicalmed6010029 (PMC8005960; doi:10.3390/tropicalmed6010029)
Supplement: Supplementary file 1 [file tropicalmed-06-00029-s001.pdf]

life

MDPI

Genetic Diversity and Drug Resistance of HIV-1 Among Infected Pregnant Women Newly Diagnosed in Luanda, Angola

DATA COLLECTION FORM (Translated from Portuguese)

1. Sociodemographic data

Age ____years
Municipality of residence in Luanda ____
Occupation____
Current level of education: ____Illiterate ____ Basic ____ Medium ____Superior
Gestational age (trimester) ____ 1st trimester ____ 2nd trimester ____ 3rd trimester ____Parturient

2. Laboratory results

Rapid Test Determine HIV 1/2
____Negative
____Positive
If TR Determine HIV 1/2 positive, perform TR UniGold HIV
____Negative
____Positive

2.1. Results of molecular analysis

Type of HIV-1 ________________
HIV-1 subtype _______________
Drug resistance mutation
____No
____Yes
If have a drug resistance mutation, which ARV class is affected
____ITRN
____ITNNN
____IP
If have a drug resistance mutation, which the resistance profile
____Low
____Intermediate
____High

3. Other information

Co-infection with any viral or bacterial etiological agent
____No
____Yes
If yes, indicate co-infection
____VHB
____VHC

____Syphilis
____TB
____Other (s), please indicate____

Date______________________ : Reseracher : _________________________
